# A Mathematical Model that Simulates Control Options for African Swine Fever Virus (ASFV)

**DOI:** 10.1371/journal.pone.0158658

**Published:** 2016-07-08

**Authors:** Mike B. Barongo, Richard P Bishop, Eric M Fèvre, Darryn L Knobel, Amos Ssematimba

**Affiliations:** 1 International Livestock Research Institute, P.O. Box 30709, Nairobi 00100, Kenya; 2 Institute of Infection and Global Health, University of Liverpool, Leahurst Campus, Neston, CH64 7TE, United Kingdom; 3 Department of Veterinary Tropical Diseases, Faculty of Veterinary Science, University of Pretoria, Pretoria, South Africa; 4 Department of Mathematics, Faculty of Science, Gulu University, P.O. Box 166, Gulu, Uganda; Hokkaido University Graduate School of Medicine, JAPAN

## Abstract

A stochastic model designed to simulate transmission dynamics of African swine fever virus (ASFV) in a free-ranging pig population under various intervention scenarios is presented. The model was used to assess the relative impact of the timing of the implementation of different control strategies on disease-related mortality. The implementation of biosecurity measures was simulated through incorporation of a decay function on the transmission rate. The model predicts that biosecurity measures implemented within 14 days of the onset of an epidemic can avert up to 74% of pig deaths due to ASF while hypothetical vaccines that confer 70% immunity when deployed prior to day 14 of the epidemic could avert 65% of pig deaths. When the two control measures are combined, the model predicts that 91% of the pigs that would have otherwise succumbed to the disease if no intervention was implemented would be saved. However, if the combined interventions are delayed (defined as implementation from > 60 days) only 30% of ASF-related deaths would be averted. In the absence of vaccines against ASF, we recommend early implementation of enhanced biosecurity measures. Active surveillance and use of pen-side diagnostic assays, preferably linked to rapid dissemination of this data to veterinary authorities through mobile phone technology platforms are essential for rapid detection and confirmation of ASF outbreaks. This prediction, although it may seem intuitive, rationally confirms the importance of early intervention in managing ASF epidemics. The modelling approach is particularly valuable in that it determines an optimal timing for implementation of interventions in controlling ASF outbreaks.

## Introduction

African swine fever (ASF) is a devastating disease in domestic pigs caused by a DNA virus of the *Asfarviridae* family [1,2]. This disease is a significant constraint to pig production, causing economic losses to pig farmers and posing a threat to food security. ASF is endemic in most parts of Africa and its recent introduction into Georgia and subsequent spread to Russia and the European Union [3] renders it a global animal health problem that needs to be dealt with urgently [4]. It is a highly contagious disease transmitted by either direct contact between infected and susceptible pigs or indirectly through contact with infectious material in the environment and on fomites [5]. African swine fever virus (ASFV) is a resistant and stable virus capable of persisting in the environment and in pig products over a wide range of temperatures and pH for a prolonged period of time thereby enabling its transmission over long distances [2]. Clinical forms of the disease vary across a spectrum from peracute through acute to chronic and in some cases, apparently healthy virus carriers arise [6]. Peracute and acute syndromes are characterised by high fever, loss of appetite, haemorrhages and cyanosis on the skin and internal organs with mortality rates of up to 100% in naïve pig herds [2,4,7,8].

ASF has no cure or vaccine and its control depends on proper use of biosecurity measures, pig confinement and movement restriction plus culling of pigs on infected farms and in surrounding areas [9]. However, movement restriction is challenging to be effectively implemented in developing countries due to limited funding for public veterinary services. Likewise, pig confinement is not widely used in resource-poor countries where a large number of pigs are free-ranging due to limited access to and high cost of quality feeds. For other livestock diseases, vaccination is a key component of control strategies. In the case of ASF, research to develop and test vaccines is ongoing and a few experimental vaccines are promising candidates but need wider evaluation before being commercialised [10–12]. In the absence of vaccines and/or chemotherapy and a lack of funds to compensate farmers in the event of culling, enhanced biosecurity remains the main ASF control measure in resource-poor countries.

In these resource-poor countries, there is limited information about animal movement patterns, factors that favour persistence of transmissible virus as well as the role of farmer behaviour in maintaining the endemic status of the disease. These factors, together with the limited knowledge about the disease’s transmission pathways renders the design of improved ASF control strategies even more difficult [13].

Mathematical models may provide insight into the epidemiology of infectious diseases and the design of control strategies. They can be used to guide the identification of critical intervention points aimed at minimising disease-related mortality (hereafter referred to as disease burden) [14]. In addition, they can be used as tools for quantifying the magnitude, duration and cost of disease epidemics [15]. Models also provide an environment to assess how interventions may change the dynamics of the disease and how benefits may accrue from these interventions [14,16]. Therefore, integrating mathematical modelling benefits the design of ASF control strategies. In this study, we develop and parameterise a mathematical model to simulate the transmission of ASFV. We use the model to assess the relative impact of different intervention scenarios as well as to determine the optimum response time to suspected ASF epidemics.

Due to data limitation, the scope of the current study was limited to simulations with the aim of using the outcomes to inform further studies. For example, the outcomes from this study provide a means to; 1) guide the design of the required experimental studies and, 2) help improve field data collection during future epidemics. In a commensurate interaction, results from these studies will in turn further refine future modelling attempts.

## Materials and Methods

### Geographical study area

The pattern of ASF outbreaks in Eastern Africa is different from that reported in Eastern Europe. In our study area some ASF outbreaks have been confirmed in areas with no wild pigs or argasid ticks (R. Bishop and E. Okoth, unpublished). Wilkinson [17] & OIE [7] have reported cases of pigs that survive ASF infection to become persistently infected (i.e., appear healthy while still shedding the ASFV virus) but when stressed they reactivate to infectious state.

The production system in the study area is characterised by low input pig husbandry practices where pigs are mainly free ranging and occasionally tethered [18]. Pigs in this kind of production system are known to cover an area within a radius of about 3km per day scavenging for food [19]. We therefore assume that pigs are homogeneously mixing due to the wide area they cover per day. Our study unit was a Parish consisting of 9 villages. This unit covers a geographical area of over 20 square Kilometers.

### Model formulation and assumptions

**Fig 1:**
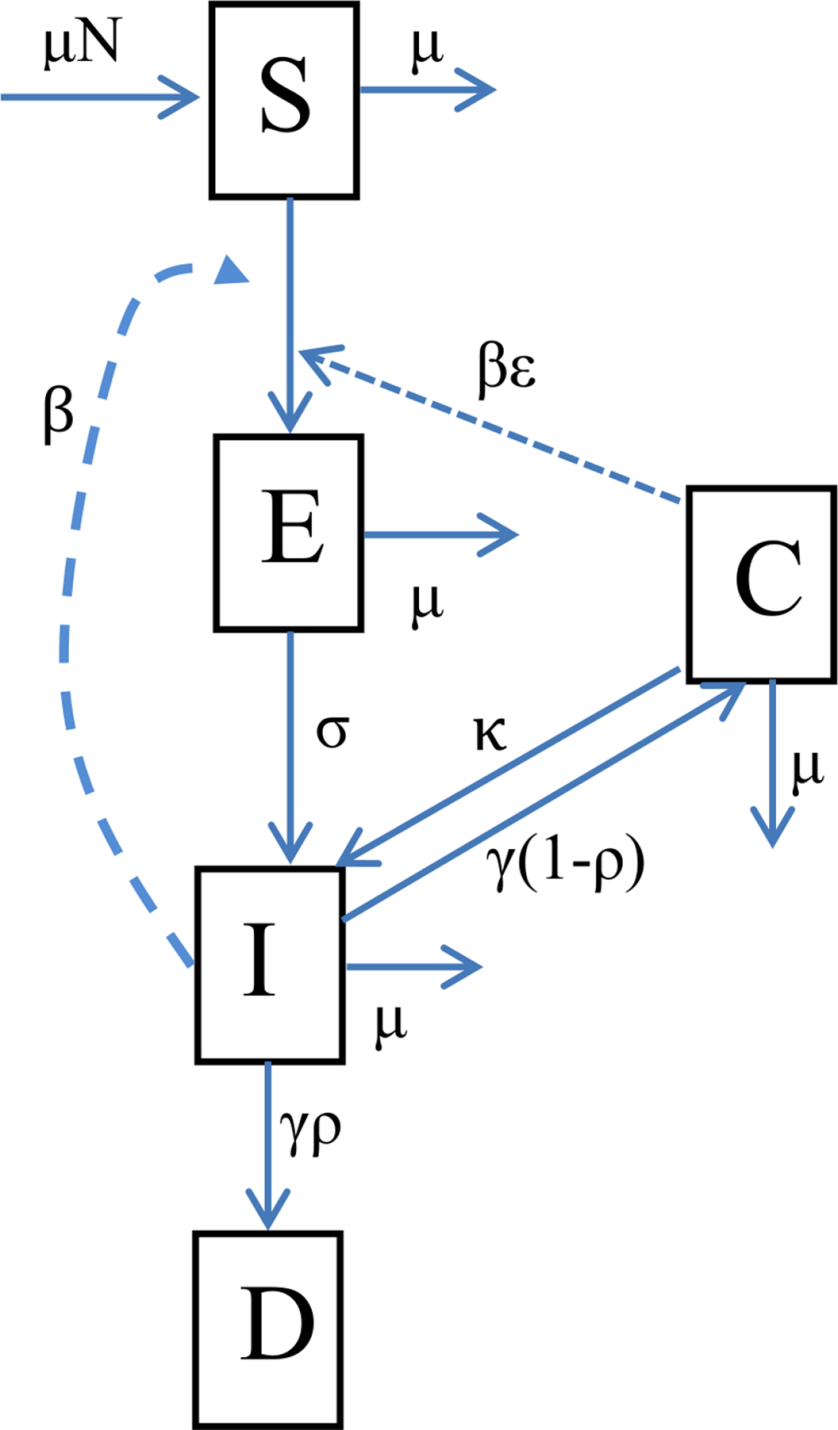
The schema shows the transition pathways between epidemiological classes of the ASF model. The transition from class (S) to class (E) was governed by transmission rate (*β*) while the transition from class (E) to class (I) was dependent on latent period (*σ*). The infectious animals either die at a rate (*γρ*) and enter class (D) or enter the carrier class (C) at a rate *γ*(1−*ρ*). Carriers also transmit at a reduced rate (*εβ*) and can re-activate to infectiousness at a rate (*κ*). There is natural mortality that occurs in each class at a rate μ. New recruits enter the S class at a rate μN.

Our model consists of five compartments categorising animals based on their status with respect to the disease: susceptible (S), infected but not yet infectious (E), infectious (I), carrier (i.e. persistently infected and asymptomatic animals, C) and the disease-induced deaths (D). The model incorporates population demographics as described by [20] and [21]. The model structure is shown in Fig 1 and events, parameter definitions, data sources and estimates are presented in Tables 1 and 2. The total population is given by *N = S + E + I + C + D*.

**Table 1:** Events defining the effect of transition between compartments and the rate at which they occur.

Event	Effect	Transition rate
Exposure	(S, E, I, C) → (S-1, E+1, I, C)	*βS*(*I* + *εC*)
Infection	(S, E, I, C) → (S, E-1, I+1, C)	*σE*
Disease mortality	(S, E, I, C) → (S, E, I-1, C)	*γρI*
Recruitment	(S, E, I, C) → (S+1, E, I, C)	*μN*
To Carrier	(S, E, I, C) → (S, E, I-1, C+1)	*γ*(1−*ρ*)*I*
Carrier reactivation	(S, E, I, C) → (S, E, I+1, C-1)	*κC*
Natural death in Susceptibles	(S, E, I, C) → (S-1, E, I, C)	*μS*
Natural death in Exposed	(S, E, I, C) → (S, E-1, I, C)	*μE*
Natural death in Infectious	(S, E, I, C) → (S, E, I-1, C)	*μI*
Natural death in Carriers	(S, E, I, C) → (S, E, I, C-1)	*μC*

**Table 2:** The Minimum, Mode and Maximum estimates used in the Pert distributions for the parameters of the model (day ^-1^).

	Definition	Min	Mode	Max	Key data source
*μ*	Non-specific mortality/ crude birth rate^*^	0.0020	0.0027	0.0035	User defined^#^
*β*	Transmission rate^$^	0.200	0.300	0.500	[26]
*γ*	ASF-specific mortality rate	0.080	0.125	0.250	[26]
*ρ*	Proportion of infectious that die	0.600	0.700	0.800	[4]
*σ*	Transition rate from exposed to infectious class	0.120	0.250	0.350	[4,7,33,34]
*κ*	Rate of reactivation of carriers^*^	0.040	0.060	0.080	[7]
*ε*	Scale-down factor on effective contact rate for carrier animals^*^	0.250	0.300	0.350	User defined

* User defined for purposes of this simulation.

^#^ Based on observed average life expectancy of 370 days.

^$^ The minimum *β* estimate of [26] is taken as the Max value for the Pert distribution in estimating *β*.

We scaled down by a factor of (1.5)^-1^ and (1.5)^-2^ respectively for the mode and minimum values.

Several assumptions are made to allow for this formulation. New animals are born into the susceptible (S) class at a constant per capita birth rate equal to the natural mortality rate *μ*. This assumption is vital to ensure that any system dynamics that we observe are likely to be disease-related. The movement of susceptible pigs from S to the Exposed (E) class is governed by the transmission rate parameter *β*. After a latent period *σ*^−1^ days, exposed pigs transit to a state of infectiousness (I). A proportion *ρ* of infectious pigs succumb to the disease while those that survive beyond the infectious period (*γ*^−1^ days) are assumed to become carrier pigs at a rate *γ*(1-*ρ*) [7,8,17,22]. Carrier pigs are also assumed to contribute to the infection pressure though at a reduced rate (*βε*) and may occasionally reactivate and transition back to the infectious class (I) at a rate *κ* [23]. Natural mortality occurs in all classes and additional disease-specific mortality occurs in the infectious class at a rate (*γρ*). We assume density-dependent transmission because the pigs freely interact and infection can occur when contact happens.

The dynamics of the system described and presented in Fig 1 are captured by the differential equations:

$$\frac{dS}{dt}=-\beta S\left(I+\varepsilon C\right)+\mu N\;-\;\mu S,$$

$$\frac{dE}{dt}=\beta S\left(I+\varepsilon C\right)-(\sigma +\mu )E,$$

$$\frac{dI}{dt}=\sigma E+\kappa C-\gamma \rho I\;-\;\gamma (1\;-\;\rho )I-\mu I,$$

$$\frac{dC}{dt}=\gamma (1-\rho )I-(\kappa +\mu )C,$$

$$\frac{dR}{dt}=\gamma \rho I$$

The system’s events were implemented stochastically using Gillespie’s direct algorithm [24,25] and 1000 simulations were run per scenario described in the section of intervention scenarios.

### Model parameters

In Table 2 we present estimates for some model parameters obtained from literature. Generally, there is limited fields and /or experimental data to quantify model parameters and those accessed varied widely. We chose to use the Pert distribution to randomly generate parameter estimates over the extracted parameter ranges. The Pert distribution is best suited to situations when information available to estimate parameters is limited but sufficient to extract the Minimum, Maximum and Mode (i.e., most likely) estimate. The non-specific mortality (*μ*) was estimated as the reciprocal of the mean life expectancy of pigs in the study region (i.e. 280–500 days). The transmission rate parameter *β(t)* has been estimated from the literature [26] taking into account the difference in pig interactions between those under experimental conditions and those in the natural setting. The scale-down factor (*ε*) on the transmission rate for carrier animals and the rate of reactivation of carriers (*κ*) to infectious state are user-defined for simulation purposes. These parameters have been set to values between zero and one due to lack of information on them pending further studies to give them appropriate values. For purposes of our exploration we set them in a low range of 0.3 and 0.06 respectively.

### Intervention scenarios modelled

Using scenario analysis approach, we assess the effect of different interventions on the cumulative number of pigs that succumb to ASF over a simulation period of 200 days. As a reference point for the assessment of impact of interventions, we started by simulating the dynamics of the disease in the population of 500 pigs without any intervention. Thereafter two categories of intervention scenarios were simulated, with each being initiated at various time points after the onset of the epidemic. The first category consisted of implementation of biosecurity measures which were modelled as a change in the time-dependent transmission rate parameter *β(t)* according to

$$\beta (t)=\{\begin{array}{cc} \beta_{1} & t<\tau \\ \beta_{0}+(\beta_{1}-\beta_{0})e^{-(t-\tau )} & t\geq \tau \end{array}$$

where τ is the time at which biosecurity interventions start [27]. *β(t)* is modelled to gradually reduce following an exponential decay from a baseline value *β*_*1*_ to a value that asymptotically approaches *β*_*0*_ (set to 0.05 in this study) [27,28]. The value of *β*_*0*_ can be set to zero if the biosecurity measures put in place are perceived to be able to stop all further transmission.

The second category of interventions modelled the potential effect of using hypothetical vaccines with varying efficacies and coverage. The vaccine-protected proportion is obtained from the product of vaccine efficacy and coverage levels. In this study, we modelled vaccination at three protection levels; 30%, 50% and 70%. Vaccination was modelled as a single pulse event during the course of the simulation. The effect of time to intervention was assessed by implementing the above interventions at 14, 30 and 60 days after the start of the epidemic. As examples the naming of the intervention scenarios was as follows: “Vac_7030” is vaccine intervention with effect of 70% and day 30 after onset of epidemic whereas “Bio_30” is a biosecurity intervention 30 days after the onset of the epidemic. Baseline is an intervention-free scenario while “Bio_Vac_7014” is a combination of biosecurity and vaccination strategy (with 70% effect) at day 14 and “Ctns_Vac_7014” is an intervention scenario where 70% are effectively vaccinated at day 14 and all new recruits thereafter are also vaccinated.

We present results of a few of the many permutations of intervention scenarios that could be compared. Day 14 and day 60 were of particular interest because they represented the earliest practical date to implement an intervention and a representation of a date not very long into the complete course of an outbreak, yet too late for an intervention to cause the desired mitigating effect. The model simulations were run in Wolfram Mathematica 9.

## Results

**Fig 2:**
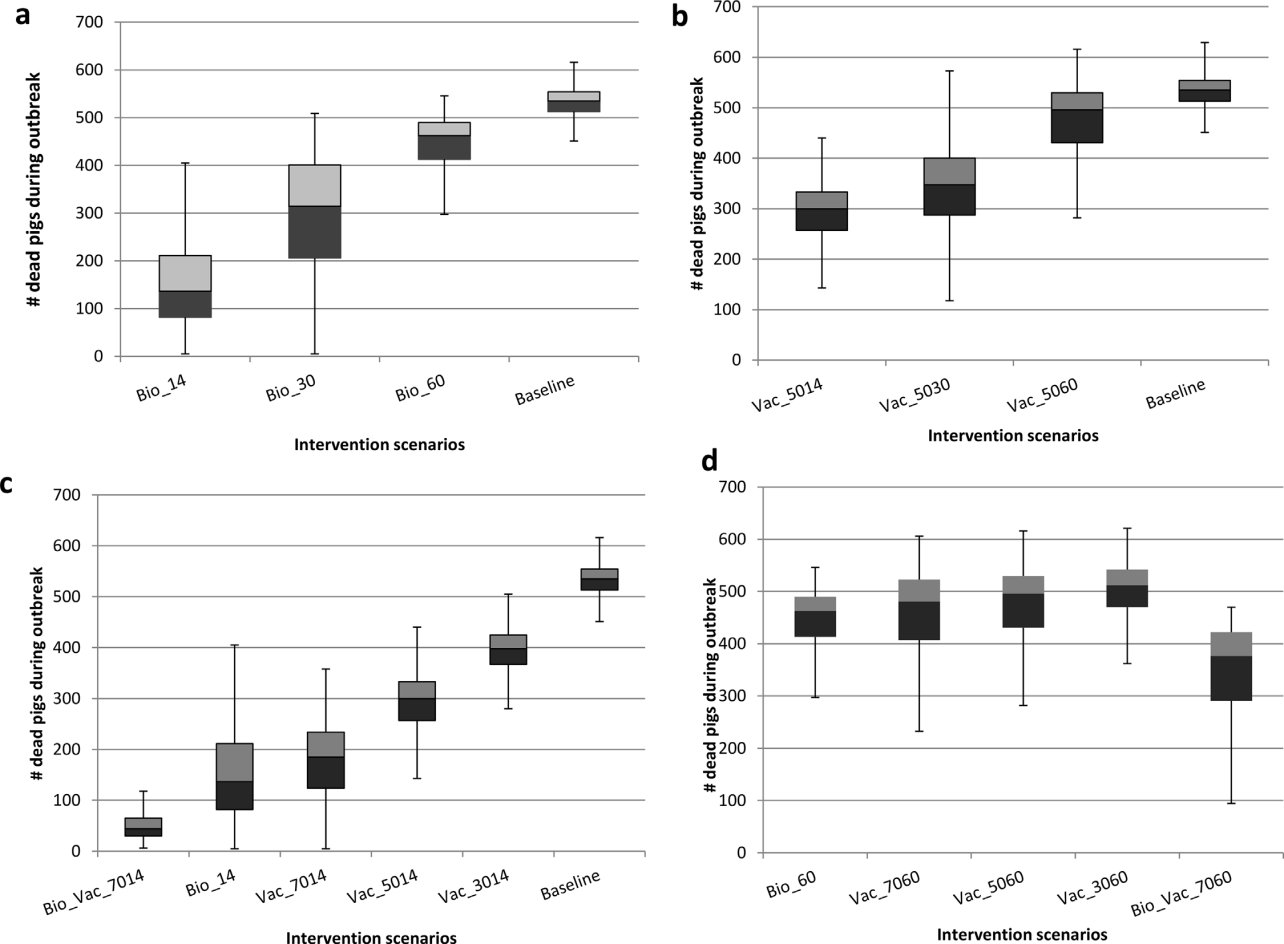
Box plots showing the effect of timing of introduction of different intervention scenarios on disease burden. The baseline box represents an intervention-free scenario. Panel (a) shows the effect of the timing of introduction of biosecurity measures after the onset of the epidemic (where Bio_xx = Biosecurity strategy implemented at day xx). Panel (b) depicts effects of vaccination (protecting 50%) implemented at day 14, 30 and 60 days on disease burden (i.e. Vac_50xx = Vaccination conferring 50% protection at day xx). Panel (c) compares the effects of different vaccine efficacies and a combination intervention strategy on disease burden when intervention is started at day 14 (Bio_Vac_7014 = Combination of biosecurity and 70% Vaccine efficacy implemented at day 14). Panel (d) depicts the effects of delayed intervention on disease burden across different strategies of vaccine efficacies and combination scenarios (i.e. Vac_yyxx = Pulse Vaccination of efficacy yy% implemented at day xx while Bio_Vac_7060 is a combination strategy of Biosecurity measures and 70% efficacy vaccine implemented at day 60).

**Fig 3:**
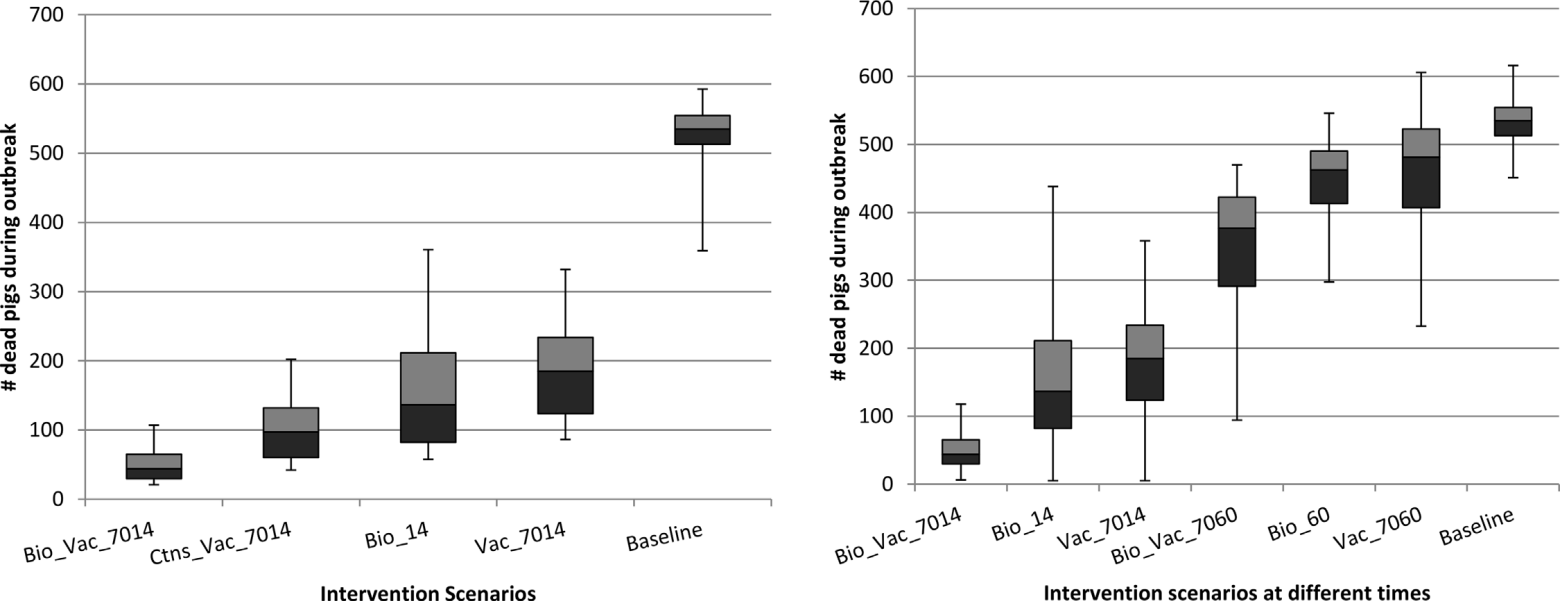
Box plots comparing different intervention scenarios at day 14 and day 60. Panel (a) shows relative impact of the intervention scenarios at day 14. Bio_Vac_7014 is a combination of biosecurity measures and vaccination with 70% effect at day 14. Box Ctns_Vac_7014 is a scenario of pulse vaccination at day 14 followed by continuous vaccination programme of new recruits. Panel (b) compares the effects similar intervention scenarios at day 14 and day 60 to show the effect of timing of intervention on disease burden irrespective of the strategy implemented.

Model predictions of the impact of the different intervention strategies on the cumulative number of pigs that succumbed to the disease are presented. The results are presented as boxplots depicting the median, lower and upper quartiles of predicted disease burden from the 1000 simulations per intervention scenario. Fig 2 depicts the disease burden for different times of introduction of intervention scenarios while Fig 3 presents a comparison of the potential impact of delaying the start of intervention strategies (scenarios for day 14 and day 60).

### Effect of enhanced biosecurity measures on the disease burden

In Fig 2A, the model predicts a 74% reduction on the disease burden if biosecurity measures are implemented 14 days after the onset of the epidemic (Bio_14) compared to the baseline scenario which predicts a median of 535 fatalities. Implementing biosecurity measures 30 and 60 days after the onset of the epidemic decreases the disease burden by 41% and 13.5% from the baseline scenario, respectively.

### Effect of vaccination interventions on the disease burden

Fig 2B shows the disease burden under pulse vaccination where 50% of the animals at risk are vaccine-protected at 14, 30 or 60 days after onset of the epidemic. Vaccinating 50% after 14 days of the epidemic onset reduced the burden by 44% while waiting for 60 days reduced the burden by 16%. Fig 2C shows the impact of the different proportions protected by the vaccine when intervening at day 14, either singly or in combination with biosecurity measures. The model predicts a 65% reduction in cumulative pig deaths when the vaccine protects 70% of the animals at risk (i.e., Vac_7014). With a delayed intervention, i.e. when intervening at day 60 post epidemic onset, there is a minimal reduction (ranging from 4% to 14%) in the disease burden across all simulated intervention scenarios (Fig 2D). Among the simulated vaccine intervention scenarios, Vac_7014 is predicted to avert the highest number of pig deaths (only 185 deaths) compared to the baseline scenario.

In Fig 3A we compare the impact of interventions implemented at day 14 involving protection of 70% of the pigs at risk through vaccination under different vaccination and biosecurity schemes. An intervention scenario involving a pulse vaccination at day 14 coupled with a continuous vaccination (and protection) of 70% of the new recruits (i.e., Ctns_Vac_7014) reduces the disease burden by 82%. A strategy combining intensified biosecurity measures and pulse vaccination and protecting 70% of the pigs at risk (i.e., Bio_Vac_7014) could avert up to 91% of pig deaths, yet the same strategy, when implemented after 60 days post epidemic onset could only save 30% (Fig 3B).

## Discussion

ASF continues to be a major constraint to the growth of the pig industry in sub-Saharan Africa and poses a significant risk to established pig industries in the developed world mainly the European Union and China. There is a need to continuously refine and update both our knowledge of its epidemiology and control measures. In the present study, we used a stochastic compartmental mathematical model to assess the potential impact of different intervention scenarios on the disease burden.

In the absence of treatment or vaccines against ASF, control strategies primarily rely on biosecurity measures [6,9]. It has been noted that smallholder pig farmers find it difficult to fully comply with biosecurity measures for a prolonged period of time because of the nature of their production systems [18]. We investigated the benefits of compelling small holder farmers to adopt and intensify biosecurity measures specifically in times of ASF outbreaks, in order to minimize the disease burden. When compared to the baseline scenario of not intervening at all, our model predicts that if biosecurity measures are enhanced within a fortnight of an epidemic onset, the disease burden can be reduced by up to 74%. This finding emphasises the need to hasten the intensification of biosecurity measures in the event of suspicion. This can be achieved through measures such as improved hygiene, isolation of sick or new pigs, movement control, treatment of swill, use of disinfectants, proper housing and disposal of dead pigs as soon as ASF is suspected [18]. This is achievable if resources are available to implement this intervention however if left entirely in the hands of farmers, they may not have the resources or incentive to meet these costs. Above all, farmers in the settings in question need to be highly aware of ASF and of the tools available to them to quickly upscale their on-farm biosecurity. This is in agreement with standard ASF control protocols, which emphasises that biosecurity is essential in the control of ASF [4,29].

In addition, we modelled three protection levels of hypothetical vaccines and three intervention time points for their use. The greatest vaccine impact (of 65% reduction in disease burden) was predicted at the highest simulated vaccine protection level of 70% when implemented at day 14 post epidemic onset. The impact of this pulse vaccination on disease burden is likely to be affected by the continuous influx of susceptible new recruits that enter the system. In an ideal situation, vaccination schemes should be designed in such a way as to include newly-recruited animals on a continuous basis. We capture this scenario by simulating interventions where vaccination is continuous and conclude that continuous vaccination reduces the disease burden by 82%.

The most effective simulated intervention strategy (with 91% of deaths averted) is a combination of pulse vaccination (protecting 70% of the pigs at risk) together with enhanced biosecurity measures implemented by day 14. However, vaccines are still a long way from being commercially available, let alone accessible and affordable to the rural pig farmers. Early attempts to develop conventional vaccines against ASFV achieved partial protection or could not be scaled up for commercial production [11]. Nonetheless, these results, although theoretical at this point, illustrate the potential impact of vaccines on disease burden and how they could improve control efficacy when combined with biosecurity measures. They also help in identifying the levels of protection that any eventual vaccine would need to attain in order to be effective in preventing epidemics.

The predicted effect of intervention strategies on the disease burden was found to be dependent on time to intervention with delayed intervention reducing the impact of intervention scenarios. For example, intervening 60 days post epidemic onset reduced the impact of all scenarios, with only 4% to 30% of baseline deaths averted as compared to reductions of 65% to 91% when intervening at day 14. This prediction, although intuitive, emphasises the importance of early intervention in managing ASF epidemics, and our modelling approach provides a means to determine appropriate and feasible intervention moments in controlling ASF, in this case found to be 14 days post epidemic onset.

ASFV varies in virulence with some strains causing 100% mortality while less virulent ones allow some pigs to recover from either sub-acute or chronic infections to become persistently infected or carrier pigs. These carrier animals are assumed to play a role in maintaining the disease in the domestic cycle and pose a major challenge to its control [7,30]. However, there is no sufficient evidence to quantify the contribution of carrier pigs to infection pressure and what proportion reactivate to an infectious state.

We recommend that further studies be carried out to more reliably quantify these model parameters using empirical data from field activities. Our studies [22,31] on ASFV p72 genotypes IX and X in East African smallholder systems indicates that transmission data is particularly hard to collect in the field with the currently available techniques, since anthropogenic effects (rapid selling to butchers, or pig farmers in distant villages) complicate collection and interpretation of transmission data. It is also difficult to detect (with the currently available tests) the genotype IX virus in blood samples by either serology or real time PCR because of great viral genetic and antigenic diversity [32]. In this study we have relied on using random parameter choice based on the Pert distribution informed by available data to improve reliability of the study’s outcomes. We envisage that in future projects involving appropriately designed experimental infections will be used to refine ASFV transmission parameters.

Although our model predicts a combination of vaccination and enhanced biosecurity as the best intervention scenario, the only currently feasible strategy is implementation of enhanced biosecurity measures. We therefore recommend intensification of active surveillance and use of pen-side diagnostic assays for rapid detection and confirmation of ASF to allow for timely implementation of enhanced biosecurity. However, we also recommend continued research on the development of a vaccine against ASFV to allow for deployment of a hybrid intervention strategy. Most importantly, given the importance of the time to implementing biosecurity measures, we recommend that veterinary services in ASF outbreak risk areas work to educate farmers on the most feasible biosecurity measures to adopt in a time efficient manner [18]. Finally, we also suggest that further work on cost-benefit analyses should be performed to compare the simulated interventions from an economic perspective.

## Supporting Information

S1 Data File containing simulation data that was used in this manuscript. (XLSX)
